# Supplementary material for: Association between Pb, Cd, and Hg Exposure and Liver Injury among Korean Adults
Source: Int J Environ Res Public Health. 2021 Jun 24;18(13):6783. doi: 10.3390/ijerph18136783 (PMC8297092; doi:10.3390/ijerph18136783)
Supplement: Supplementary file 1 [file ijerph-18-06783-s001.zip › ijerph-1261416-supplementary.pdf]

Supplemental Material, Table S1. The descriptive statistics of demographic characteristics and liver function biomarkers concentration GM (95% CI) in participants.

	N (%)	AST	ALT	GGT
**Total**	2,953 (100)	22.45 (22.16-22.74)	19.26 (18.93-19.60)	18.02 (17.51-18.55)
**Sex**				
Male	1205 (40.81)	23.18 (22.76-23.61)	21.97 (21.38-22.59)	24.05 (23.14-25.00)
Female	1748 (59.19)	21.82 (21.51-22.13)	17.16 (16.81-17.51)	13.97 (13.53-14.43)
p-value		<0.001	<0.001	<0.001
**Age group(years)**				
19-29	231 (7.82)	20.69 (20.05-21.34)	17.42 (16.60-18.29)	15.85 (14.67-17.14)
30-39	411 (13.92)	21.17 (20.65-21.70)	18.77 (17.98-19.59)	17.31 (16.33-18.36)
40-49	500 (16.93)	22.14 (21.58-22.71)	19.57 (18.87-20.30)	18.31 (17.24-19.45)
50-59	680 (23.03)	23.57 (23.14-24.01)	20.72 (20.03-21.44)	19.94 (19.10-20.82)
60-69	712 (24.11)	24.25 (23.80-24.69)	20.74 (20.17-21.33)	19.20 (18.54-19.88)
≤ 70	419 (14.19)	24.25 (23.74-24.76)	18.67 (18.03-19.33)	17.97 (16.91-19.10)
p-value		<0.001	<0.001	<0.001
**BMI(kg/m^2)**				
< 18.5	74 (2.51)	20.80 (19.39-22.32)	15.57 (14.15-17.14)	13.54 (11.77-15.56)
18.5-23	996 (33.73)	21.75 (21.36-22.15)	17.07 (16.63-17.52)	14.94 (14.36-15.54)
23-25	771 (26.11)	22.60 (22.08-23.12)	19.39 (18.84-19.95)	18.29 (17.40-19.22)
> 25	1,112 (37.66)	23.25 (22.92-23.60)	22.22 (21.60-22.86)	22.31 (21.42-23.24)
p-value		<0.001	<0.001	<0.001
**Smoking status**				
Never	1,974 (66.85)	22.05 (21.74-22.35)	18.14 (17.78-18.50)	15.26 (14.80-15.73)
Former	553 (18.73)	23.86 (23.34-24.40)	21.99 (21.24-22.77)	23.70 (22.50-24.97)
Current	426 (14.43)	22.55 (21.94-23.17)	21.06 (20.18-21.98)	25.37 (23.70-27.15)
p-value		<0.001	<0.001	0.005
**Drinking status**				
Never	1,003 (33.97)	22.52 (22.11-22.94)	18.77 (18.26-19.29)	15.50 (14.93-16.10)
Light	1,020 (34.54)	22.15 (21.69-22.61)	19.08 (18.50-19.69)	16.05 (15.31-16.82)
Heavy	930 (31.49)	22.69 (22.29-23.10)	19.84 (19.28-20.42)	22.67 (21.54-23.86)
p-value		0.016	0.001	<0.001
**Fish consumption frequency**				
Rarely	287 (9.72)	22.07 (21.46-22.71)	18.24 (17.36-19.16)	16.77 (15.51-18.13)
Sometimes	2,469 (83.61)	22.46 (22.13-22.79)	19.34 (18.99-19.71)	18.13 (17.55-18.74)
Often	197 (6.67)	23.00 (22.37-23.64)	20.15 (19.04-21.32)	18.92 (17.48-20.48)
p-value		0.091	0.003	0.021
**HDL-Cholesterol (mg/dL)**				
HDL < 60	1,925 (65.19)	22.55 (22.20-22.91)	20.34 (19.88-20.81)	19.68 (19.08-20.29)
HDL ≥ 60	1,028 (34.81)	22.28 (21.93-22.62)	17.65 (17.24-18.07)	15.64 (14.93-16.40)
p-value		0.098	<0.001	<0.001

[a]AST < 34 U/L, serum ALT 10–49 U/L, serum GGT < 73 U/L (male), and serum GGT < 38 U/L (female).
[b]Total mercury
Abbreviations: AST, alanine aminotransferase; ALT, aspartate aminotrans ferase; GGT, gamma-glutamyl transferase; BMI, body mass index, HDL, high-density lipoproteins cholesterol

Supplemental Material, Table S2. Adjusted proportional changes (95% CI) between heavy metals and whole range of liver function biomarkers (n=3,699).

		AST	ALT	GGT
	**N**	**Exp β (95% CI)**	**Exp β (95% CI)**	**Exp β (95% CI)**
**BPb (μg/dL)**				
Q1 (0.33-1.26)	922	1.000 (Reference)	1.000 (Reference)	1.000 (Reference)
Q2 (1.26-1.71)	927	1.031 (0.98-1.08)	1.038 (0.97-1.11)	0.980 (0.90-1.06)
Q3 (1.71-2.31)	925	1.010 (0.98-1.05)	1.010 (0.95-1.07)	1.033 (0.95-1.13)
Q4 (2.31-20.58)	925	1.071 (1.03-1.12)	1.062 (1.00-1.13)	1.185 (1.08-1.30)
p for trend		0.005	0.124	<0.001
**BHg(μg/L)**				
Q1 (0.33-1.86)	924	1.000 (Reference)	1.000 (Reference)	1.000 (Reference)
Q2 (1.86-2.81)	925	1.034 (0.99-1.08)	1.110 (1.05-1.17)	1.097 (1.028-1.170)
Q3 (2.81-4.42)	924	1.046 (1.00-1.10)	1.133 (1.06-1.21)	1.177 (1.089-1.273)
Q4 (4.43 -125.49)	926	1.058 (1.01-1.10)	1.171 (1.10-1.25)	1.257 (1.169-1.353)
p for trend		0.012	<0.001	<0.001
**UHg (μg/L)**				
Q1 (0.10-0.23)	939	1.000 (Reference)	1.000 (Reference)	1.000 (Reference)
Q2 (0.24-0.34)	892	1.009 (0.972-1.048)	1.023 (0.966-1.085)	1.012 (0.950-1.078)
Q3 (0.35-0.63)	932	1.019 (0.976-1.064)	1.046 (0.993-1.103)	1.040 (0.965-1.122)
Q4 (0.64-8.70)	936	1.028 (0.989-1.069)	1.071 (1.008-1.138)	1.104 (1.008-1.210)
p for trend		0.16	0.027	0.022
**UCd (μg/L)**				
Q1 (0.05-0.23)	922	1.000 (Reference)	1.000 (Reference)	1.000 (Reference)
Q2 (0.23-0.49)	926	1.010 (0.975-1.047)	1.046 (0.993-1.103)	1.030 (0.956-1.112)
Q3 (0.49-0.90)	927	1.012 (0.966-1.062)	1.082 (1.021-1.148)	1.094 (0.010-1.185)
Q4 (0.90-20.49)	924	1.052 (1.006-1.101)	1.127 (1.059-1.202)	1.156 (1.041-1.284)
p for trend		0.065	<0.001	0.007

[a]Total mercury

Adujsted for sex, age, smoking status, drinking status, BMI, fish consumption frequency, and HDL

Abbreviations: BPb,blood lead; BHg, blood mercury; UCd, urinary cadmium; AST, alanine aminotransferase; ALT, aspartate aminotrans ferase; GGT, gamma glutamyl transferase

Supplemental Material, Table S3. Heavy metal-related occupation-adjusted proportional changes (95% CI) of liver function biomarkers by heavy metals exposure (n=2,953).

		AST[a]	ALT[a]	GGT[a]
	N	Exp β (95% CI)	Exp β (95% CI)	Exp β (95% CI)
**BPb (μg/dL)**				
Q1 (0.33-1.26)	737	1.000 (Reference)	1.000 (Reference)	1.000 (Reference)
Q2 (1.26-1.71)	739	1.021 (0.994-1.049)	1.047 (1.007-1.089)	1.016 (0.963-1.073)
Q3 (1.711-2.31)	738	1.023 (0.997-1.050)	1.029 (0.986-1.075)	1.066 (1.004-1.133)
Q4 (2.31-14.82)	739	1.040 (1.011-1.073)	1.047 (1.000-1.095)	1.095 (1.029-1.166)
p for trend		0.007	0.108	0.001
**BHg[b] (μg/L)**				
Q1 (0.33-1.86)	738	1.000 (Reference)	1.000 (Reference)	1.000 (Reference)
Q2 (1.86-2.81)	735	1.027 (1.013-1.051)	1.053 (1.013-1.096)	1.010 (0.961-1.062)
Q3 (2.81-4.42)	742	1.010 (0.985-1.036)	1.050 (0.012-1.091)	1.073 (1.017-1.131)
Q4 (4.43-125.49)	738	1.044 (1.016-1.073)	1.119 (1.075-1.164)	1.183 (1.112-1.260)
p for trend		0.009	<0.001	<0.001
**UHg[b] (μg/L)**				
Q1 (0.10-0.22)	725	1.000 (Reference)	1.000 (Reference)	1.000 (Reference)
Q2 (0.23-0.33)	764	1.013 (0.991-1.035)	1.039 (0.997-1.081)	1.044 (0.930-1.097)
Q3 (0.34-0.61)	732	0.995 (0.969-1.023)	1.014 (0.969-1.062)	1.019 (0.961-1.080)
Q4 (0.62-8.70)	732	1.003 (0.976-1.030)	1.022 (0.974-1.071)	1.045 (0.990-1.104)
p for trend		0.930	0.588	0.124
**UCd (μg/L)**				
Q1 (0.05-1.86)	739	1.000 (Reference)	1.000 (Reference)	1.000 (Reference)
Q2 (1.86-2.81)	736	1.024 (0.997-1.051)	1.045 (1.001-1.091)	1.013 (0.951-1.079)
Q3 (2.81-4.42)	741	1.008 (0.978-1.038)	1.076 (1.022-1.132)	1.048 (0.989-1.111)
Q4 (4.43-16.81)	737	1.034 (1.000-1.068)	1.191 (1.030-1.155)	1.082 (1.014-1.154)
p for trend		0.130	0.001	0.028

[a]AST < 34 U/L, serum ALT 10–49 U/L, serum GGT < 73 U/L (male), and serum GGT < 38 U/L (female)
[b]Total mercury
Adujsted for sex, age, smoking status, drinking status, BMI, fish consumption frequency, and HDL
Abbreviations: BPb, blood lead; BHg, blood mercury; UHg, urinary mercury; UCd, urinary cadmium; AST, alanine aminotransferase; ALT, aspartate aminotrans ferase; GGT, gamma glutamyl transferase
